# DNase Activity of *Prevotella intermedia* Impairs Biofilm Development and Neutrophil Extracellular Trap Formation

**DOI:** 10.1002/mbo3.70102

**Published:** 2025-10-28

**Authors:** Fumi Seto‐Tetsuo, Hiroki Ashizawa, Yuko Sasaki, Hideharu Yukitake, Mikio Shoji, Naoki Iwanaga, Hiroshi Mukae, Mariko Naito

**Affiliations:** ^1^ Department of Microbiology and Oral Infection, Graduate School of Biomedical Sciences Nagasaki University Nagasaki Japan; ^2^ Department of Respiratory Medicine Nagasaki University Graduate School of Biomedical Sciences Nagasaki Japan

**Keywords:** biofilm, DNase, neutrophil extracellular traps, periodontal disease, prevotella intermedia

## Abstract

Chronic periodotitis is caused by the formation of biofilms. *Prevotella intermedia*, a gram‐negative obligate anaerobic bacterium residing in periodontal pockets is involved in the formation of biofilms and secrets a highly potent DNA‐degrading activity. Biofilm contains extracellular DNA as a structural component, suggesting that DNase activity may influence *P. intermedia's* own biofilm development. Neutrophil extracellular traps (NETs) have mesh‐like structures and composed of DNA, histone and antibacterial proteins. NETs play an important role in protecting against infection, but it is possible that DNase of *P. intermedia* disrupts NETs. The lack of established genetic manipulation has significantly delayed the analysis of DNase pathogenic factors. Recently, we have succeeded in establishing a genetic manipulation technique for *P. intermedia*. In this study, we created strains lacking two DNase candidate genes, *nucA* (PIOMA14_I_0621) and *nucD* (PIOMA14_II_0624), that were highly conserved among *P. intermedia* strains. We examined biochemical analysis of DNase activity, their effection on biofilm formation, and their evasion of NETs. Here, we showed both of them possessed DNase activities which appeared to account all of DNase activities of the bacterium. The mutant analysis further demonstrated that NucA and NucD destroyed biofilm and NETs formations. Neither one was perfectly responsible for DNase activity, but rather they take turns depending on the conditions. In conclusion, the *nucA* and *nucD* genes encode DNases that cooperatively function on biofilm formation and suppress NETs formation in *P. intermedia*.

## Graphical abstract

*Prevotella intermedia* secretes DNases (NucA and NucD), which degrade extracellular DNA in biofilms and neutrophil extracellular traps. These activities reduce biofilm stability and impair innate immune defense, suggesting that DNase production is as a virulence mechanism and a potential therapeutic target.

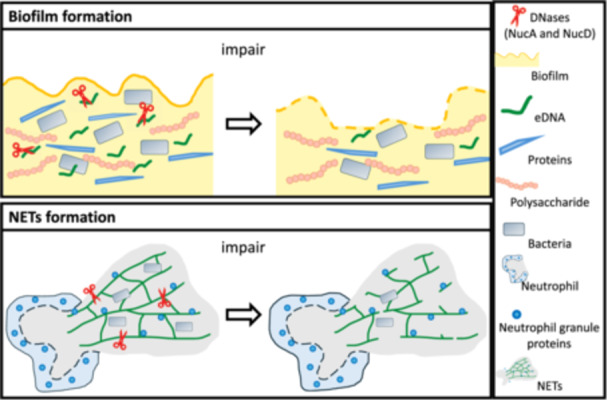

## 1 Introduction

Periodontitis is a bacterial infection disease caused mainly by dental plaque and subgingival biofilms. Oral biofilm is a complex, structured microbial community that develops through sequential colonization, microbial adhesion to tooth surfaces, and maturation within specific ecological niches of the oral cavity. An attachment is initiated by early colonizers, such as *Streptococcus species*, and mediated by various adhesive forces, including van der Waals forces, ionic interactions, quorum sensing, Brownian motion, surface tension, adhesion, and cohesion. As the biofilm matures, other bacteria, such as *Actinomycetes*, *Fusobacterium species*, and *P. intermedia* join the biofilm community. Thus, microorganisms exist in a structured and organized state, forming distinct layers based on their metabolic activity (Dewhirst et al. 2010; Gerardi et al. 2024; Verma et al. 2018). Putative periodontal pathogens are enriched as the resident oral microbiota evoke tissue destruction, thus inducing an unremitting loop of proteolysis, inflammation, and enrichment for periodontal pathogens. Microbial pathogens and sustained gingival inflammation are critical to periodontal disease progression. Tooth loss leads to occlusion, mastication, esthetics, and speech disorders, and thus, significantly reduces the patient's quality of life (Hajishengallis 2015). *P. intermedia*, which exists in the oral cavity, is a gram‐negative obligate anaerobic bacterium and one of the main pathogenic bacteria of chronic periodontitis. A characteristic of this bacterium compared to other periodontal pathogens is a strong DNA‐degrading potential (Doke et al. 2017). The virulence factors of *P. intermedia* have been reported to include LPS, protease, hemolysin, hemagglutinin, and fimbriae (Hashimoto et al. 2003; Iyer et al. 2010; Mallorquí‐Fernández et al. 2008; Sengupta et al. 2014). Recombinant proteins of various genes expected to be virulence factors were expressed in *E. coli*, purified to homogeneity, and their activities were determined, and in many cases, their three‐dimensional structures were elucidated. The potent DNA degradation activity of this bacterium has been recognized in *P. intermedia*, and the analysis expressing recombinant proteins of *P. intermedia* in *E. coli* identified and characterized DNases activities in NucA and NucD (Doke et al. 2017). The potent secreted DNases of *P. intermedia* have been shown to degrade extracellular DNA structures such as neutrophil extracellular traps (NETs) more efficiently than other Gram‐negative periodontal pathogens, suggesting a mechanism for immune evasion and enhanced pathogenicity (Doke et al. 2017). Additionally, screening of 34 periodontal bacterial species revealed that *P. intermedia* among red/orange complex pathogens exhibits particularly high levels of extracellular DNase activity, collectively supporting the hypothesis that these enzymes significantly contribute to periodontal disease progression (Palmer et al. 2012). However, an analysis to clarify the causal relationship has not been performed yet, mainly because a method for creating gene mutants that can be used with this bacterium has not been established. The purpose of this study was to elucidate the role of secreted DNase in the virulence of *P. intermedia*, particularly in biofilm and NETs formations. To achieve this, we provided a breakthrough in establishing a method for producing a mutant strain of bacterial genes for the first time. This study clarifies the presence of a pathogenic mechanism of *P. intermedia* mediated by DNases. These molecules may be a potent target molecule for suppressing periodontal disease.

## 2 Materials and Methods

### 2.1 Bacterial Strains and Culture Conditions

*P. intermedia* OMA14 strain was maintained in tryptic soy broth medium (30 g/L tryptic soy broth supplemented with 2.5 g/L yeast extract, 1 g/L cysteine, 5 μg/mL hemin and 0.5 μg/mL menadione) and TS agar plates (40 g/L tryptic soy agar base supplemented with 10 g/L of yeast extract, 1 g/L cysteine, 5 μg/mL hemin and 0.5 μg/mL menadione) under anaerobic condition. For allelic replacement studies, *P. intermedia* strains were grown in enriched BHI medium (37 g/L BHI, 2.5 g/L yeast extract, 5 μg/mL hemin and 0.5 μg/mL menadione). To prepare blood agar plates, 5% defibrinated sheep blood was added to the TS agar. For DNA agar plate assay, *P. intermedia* strains were maintained in DNA agar plates [DNA agar 42.1 g/L (Shimadzu Diagnostics Corporation, Tokyo, Japan), 5 g/L BHI, 2.5 g/L yeast extract, 5 μg/mL hemin, 0.5 μg/mL menadione, 1 mM CaCl_2_ and 1 mM MgCl_2_]. *E. coli* was maintained on LB agar plates. Antibiotics were used at the following concentrations: 100 μg/mL ampicillin (Ap; 100 μg/mL) for *E. coli* and erythromycin (Em; 10 μg/mL), gentamicin (Gm; 100 μg/mL), and cefoxitin (Cfx; 10 μg/mL) for *P. intermedia*.

### 2.2 Construction of *P. Intermedia* Mutant Strains

All strains (Supporting Information S1: Table S1) were maintained under anaerobic condition (10% CO_2_, 10% H_2_ and 80% N_2_). We used the annotated genome sequences of strain OMA14 in GenBank under Accession Number AP014597 and AP014598. The plasmids and oligonucleotides used in study are listed in Supplementary Tables S2 and S3, respectively.

**Figure 1:**
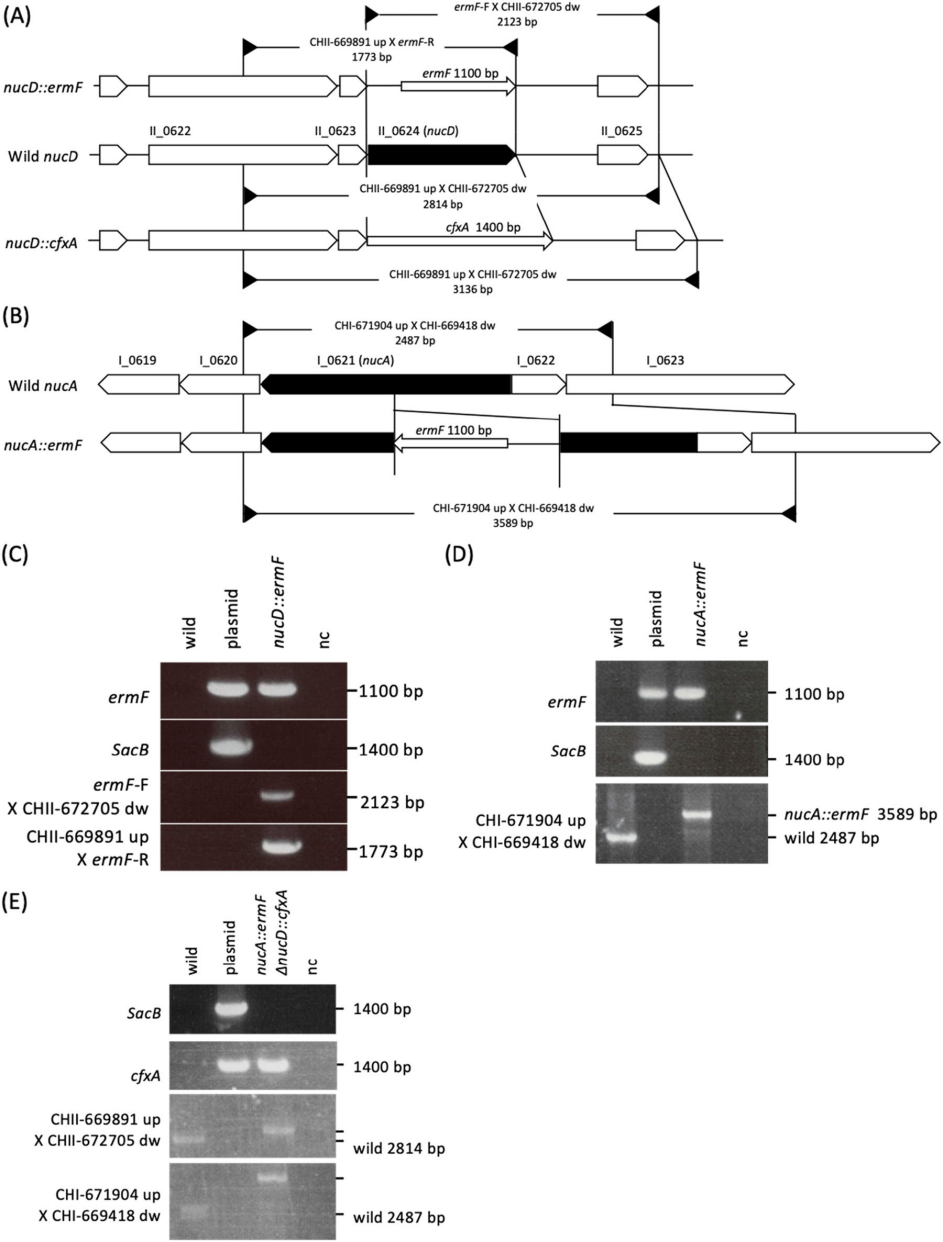
Generation of *P. intermedia* mutants. (A) and (B) Chromosomal structures at the *nucD, nucA and nucA nucD* loci on the mutant. The CDSs are depicted by arrows. Black arrows indicate target genes. Black triangles indicate PCR primers. (C)–(E) Agarose gel electrophoresis of the PCR products obtained by the primer pairs indicated left. nc: negative control (distilled water).

The targeting DNA was constructed as follows. The upstream and downstream regions of the target genes were amplified with two pairs of primers (geneX‐Up‐F/geneX‐Up‐R; geneX‐Dw‐F/geneX‐Dw‐R, where “geneX” indicates the name of the target gene and “Up,” “Dw,” “F,” and “R” indicate upstream, downstream, forward and reverse, respectively). The *ermF* region of the *ermF* DNA cassette was amplified with ErmF‐F/ErmF‐R fromppKD718. Using the three purified products, further PCR was performed with geneXUp‐F/geneX‐Dw‐R. Finally, the desired PCR products were ligated into pBSSK (pNNK0014: pBSSK *nucA::ermF*, and pDNK0015: pBSSK *nucD::ermF*). *cfxA* gene fragment was amplified using cfxA‐F plus cfxA‐R from pLYL05, then inserted into SalI‐NdeI site of pDNK0015 (pDNK0016: pBSSK *nucD::cfxA*). The BamHI‐XhoI fragment of targeting cassette was inserted into the BamHI‐XhoI site of pTCB‐*sacB* to yield conjugation plasmids for mutagenesis (pDNK17, pDNK18 and pDNK19). The resulting plasmids were transformed into *E. coli* S17‐1 and used to generate *P. intermedia* mutants by conjugal transfer and allelic replacement of the target gene with *ermF* gene, as reported previously (Naito et al. 2022). The mutant strains were designated NDP005 (*nucA::ermF*) and NDP006 (∆*nucD::ermF*). Then pDNK0019 was transformed into NDP005 to generated NDP007 (*nucA::ermF ∆nucD:: cfxA*) mutant strain, as described above. The correct deletion of the target genes was verified by PCR (see Figure 1C–E).

### 2.3 DNase Activity Assay In Vitro

DNase activities of *P. intermedia* OMA14 and its mutant strains were measured as previously reported (Doke et al. 2017). In brief, overnight culture of OMA14 and mutant strains were diluted to an optical density at 600 nm (OD_600_) value of 1.0. Therefore, bacterial supernatant was separated by centrifugation at 12,000 × *g* for 15 min. The centrifuged culture supernatant was collected and mixed with *E coli* plasmid pTCB (1 µg). The mixture (10 µL) was incubated in activation buffer containing 1 mM MgCl_2_ and 1 mM CaCl_2_ at 37°C for 15–180 min. The reaction was stopped by addition of EDTA to final concentration of 20 mM, and subjected to an agarose electrophoresis at 0.8%. The gel was added to ethidium bromide (0.1 µg/mL) and visualized under ultraviolet light.

### 2.4 Fluorescence Based Quantitative DNase Activity Assay

The fluorescence intensity based DNase activity assay was measured as previously reported (Rostami et al. 2022). An oligonucleotide probe, 5’ HEX‐CCC CGG ATC CAC CCC‐ BHQ2 3’ (PrimeTime probe Integrated DNA Technologies), was used for quantification of DNase activity. Fluorescence measurement was made by mixing of HEX‐BHQ1 substrate, diluted to 2 μM in the buffer consisting of 20 mM Tris‐HCl pH 8.0 and 10 mM CaCl_2_, with 20 μL of conditioned media. The mixture was incubated at 37°C for 30 min, and the fluorescence was measured using the Mx3005Pro (Agilent Technologies, California, CA).

### 2.5 Determination of Extracellular DNA Degradation Activity on DNA Agar Plate

DNA agar plate assay was performed according to the previous report (Morita et al. 2014) with some modifications. In brief, overnight cultures of *P. intermedia* OMA14 and mutant strains were diluted to an optical density at 600 nm of 0.5. An aliquot (5 µL) of the diluted culture was spotted onto DNA agar plate [DNA agar 42.1 g/L (Shimadzu Diagnostics Corporation, Tokyo, Japan), 5 g/L BHI, 2.5 g/L yeast extract, 5 μg/mL hemin, 0.5 μg/mL menadione, 1 mM CaCl_2_ and 1 mM MgCl_2_] and dried for 5 min. The plates were incubated at 37°C for 2 days under anaerobic conditions. The red zone appearing around bacterial colony was measured using photoshop.

### 2.6 Determination of pH Dependence of DNase Activity

To determine the optimal pH of the DNase activity, conditioned media and plasmid pTCB (1 µg) were incubated with aliquot (4 µL) of condition media of bacterial culture in 50 mM sodium acetate buffer (pH 5.0–5.4) or phosphate buffer (pH 6.0–8.0) containing 1 mM CaCl_2_ and 1 mM MgCl_2_ at 37°C for 30 min. The reaction was stopped with EDTA (final 20 mM) and the samples were subjected to an electrophoresis in 0.8% agarose gel. Plasmid DNA was stained with ethidium bromide and visualized under ultraviolet light.

### 2.7 Biofilm Formation Assay

Biofilm formation assay with crystal violet of the *P. intermedia* strains was conducted using the method reported previously (Narita et al. 2014) with some modifications. An overnight culture was diluted with fresh TS medium to yield an OD_600_ value of 0.2. Cultured media (40 g/L tryptic soy agar base supplemented with 10 g/L of yeast extract, 1 g/L cysteine, 5 μg/mL hemin and 0.5 μg/mL menadione, 1 mM CaCl_2_ and 1 mM MgCl_2_) was added to 250 µL/wells of 96‐well flat‐bottom polystyrene microtiter plates (Corning, New York, NY). After the plates were anaerobically incubated at 37°C for 24 h, planktonic cells in liquid medium were discarded and the plates were washed twice with PBS. The attached biofilms were stained with 0.1% crystal violet solution for 5 min. Then, the plates were rinsed twice with distilled water to remove excess dye and air dried. All dye associated with the attached biofilms was dissolved with 200 μL of 100% ethanol for 5 min, and then OD_540_ absorbance was measured by use of Model 680 microplate reader (BioRad, Carfornia, CA) to determine the amount of biofilm formation.

eDNA production in biofilm was quantified with PicoGreen (Invitrogen, MA, USA) using the method reported previously (Tang et al. 2013). An overnight culture was diluted with fresh TS medium to yield an OD_600_ value of 0.2. Cultured media (40 g/L tryptic soy agar base supplemented with 10 g/L of yeast extract, 1 g/L cysteine, 5 μg/mL hemin and 0.5 μg/mL menadione, 1 mM CaCl_2_ and 1 mM MgCl_2_) was added to 250 µL/wells of 96‐well flat‐bottom polystyrene microtiter plates (Corning, New York, NY). After the plates were anaerobically incubated at 37°C for 24 h, planktonic cells in liquid medium were discarded and the plates were washed twice with PBS. The attached biofilms were collected TE buffer and added PicoGreen solution. Wells with PicoGreen were incubated for 5 min before measuring the fluorescence intensity (extraction 485 nm/emission 535 nm) using a fluorescence plate reader (Beckman Coulter, California, CA).

### 2.8 Purification of Murine Neutrophil and NETs Incubation

Male C57BL/6 J mice were purchased from Jackson Laboratory, Japan. Mice were maintained at the Research Center for Biomedical Models and Animal Welfare, Nagasaki University Graduate School of Biomedical Sciences. This study was approved by Nagasaki University (2109091745), and all animal experiments were performed in accordance with the guidelines of the Research Center for Biomedical Models and Animal Welfare at Nagasaki University. Eight‐week mice were intraperitoneally administered 4% thioglycolate. After 18 h, intraperitoneal lavage fluid was collected using PBS containing 0.1% gelatine. Diff‐Quik staining confirmed that almost all cells present in the intraperitoneal lavage fluid were neutrophils. To allow to attach the neutrophils to the glass slide bottom (Thermo Fisher, MA, USA), glass slides were coated with ε‐poly‐_L_‐lysine (Cosmo Bio, Japan) for 1 h at 37°C. After PBS wash, for visualization of NETs degradation, 5.0 × 10^5^ cells were seeded on a coated glass slide and incubated for 30 min to allow the cells to attach to the glass slide bottom. For quantification of NETs degradation, 1.0 × 10^5^ cells were seeded on a coated 96‐well plate and incubated for 30 min to allow the cells to attach to the well bottom. For inducing NETs, we used a previously reported method (Doke et al. 2017) was employed with some modifications. Cells were stimulated with 100 nM phorbol 12‐myristate 13‐acetate (PMA) (Nakarai Tesque, Kyoto, Japan) and incubated for 3 h.

### 2.9 Visualization of NETs Degradation

After NETs incubation, the medium was replaced with nuclease assay buffer composed of 20 mM HEPES (pH 7.0), 150 mM NaCl, 1 mM CaCl_2_, and 1 mM MgCl_2_. Then bacterial supernatants were added at a ratio of 1:100. After 3 h, glass slides were washed with PBS and fixed with 4% paraformaldehyde for 15 min. Then, Sytox Green Nucleic Acid Stain (Invitrogen, MA, USA) was performed according to the manufacture's protocols. NETs were observed under a fluorescence microscope (Zeiss Axio Observer: Carl Zeiss, Jena, Germany).

### 2.10 Quantification of NETs Degradation

After NETs incubation, the medium was replaced with nuclease assay buffer composed of 20 mM HEPES (pH 7.0), 150 mM NaCl, 1 mM CaCl_2_, and 1 mM MgCl_2_. Then bacterial supernatants were added at a ratio of 1:100. Neutrophil elastase released in the reaction buffer in accordance. After 3 h, NETs degradation was quantified using the Mouse Neutrophil Elastase/ELA2 Immunoassay kit (R&D Systems, Inc. MN, USA).

### 2.11 Statistical Analysis

All experiments were repeated at least three times, with two or more replicates per experiment. Comparisons between two groups were analyzed for statistical significance using unpaired Student's *t*‐tests. One‐way analysis of variance (ANOVA) followed by Tukey's HSD was used for other statistical analysis (GraphPad Prism version 7.0, GraphPad Software, San Diego, CA, USA). Information regarding exact sample sizes (*n*), summary, and *P*‐value are provided in Supporting Information S1: Table S4.

## 3 Results

### 3.1 Targeted Mutagenesis in *P. Intermedia* OMA14 to Generate NucA, NucD and NucA NucD Strains

Two DNases of *P. intermeida*, NucA and NucD, were biochemically characterized using recombinant forms expressed in *E. coli* (Doke et al. 2017). To elucidate how these DNases are working in vivo, we created single mutant strains, *nucA* and *nucD*, and a double mutant strain, *nucA nucD*. First, we succeeded in creating *nucD::ermF* strain. Internal portion of *nucD* gene was replaced with *ermF* in the same gene direction (Figure 1A). The resulting mutants were examined for genome organization by PCR (Figure 1C). We finally created *nucA::ermF* and ∆*nucD::cfxA* strain (Figure 1A,B). The resulting mutants were comfirmed by genome organization by PCR (Figure 1D,E).

### 3.2 Characterization of *P. Intermedia* OMA14 Mutant Strains

**Figure 2:**
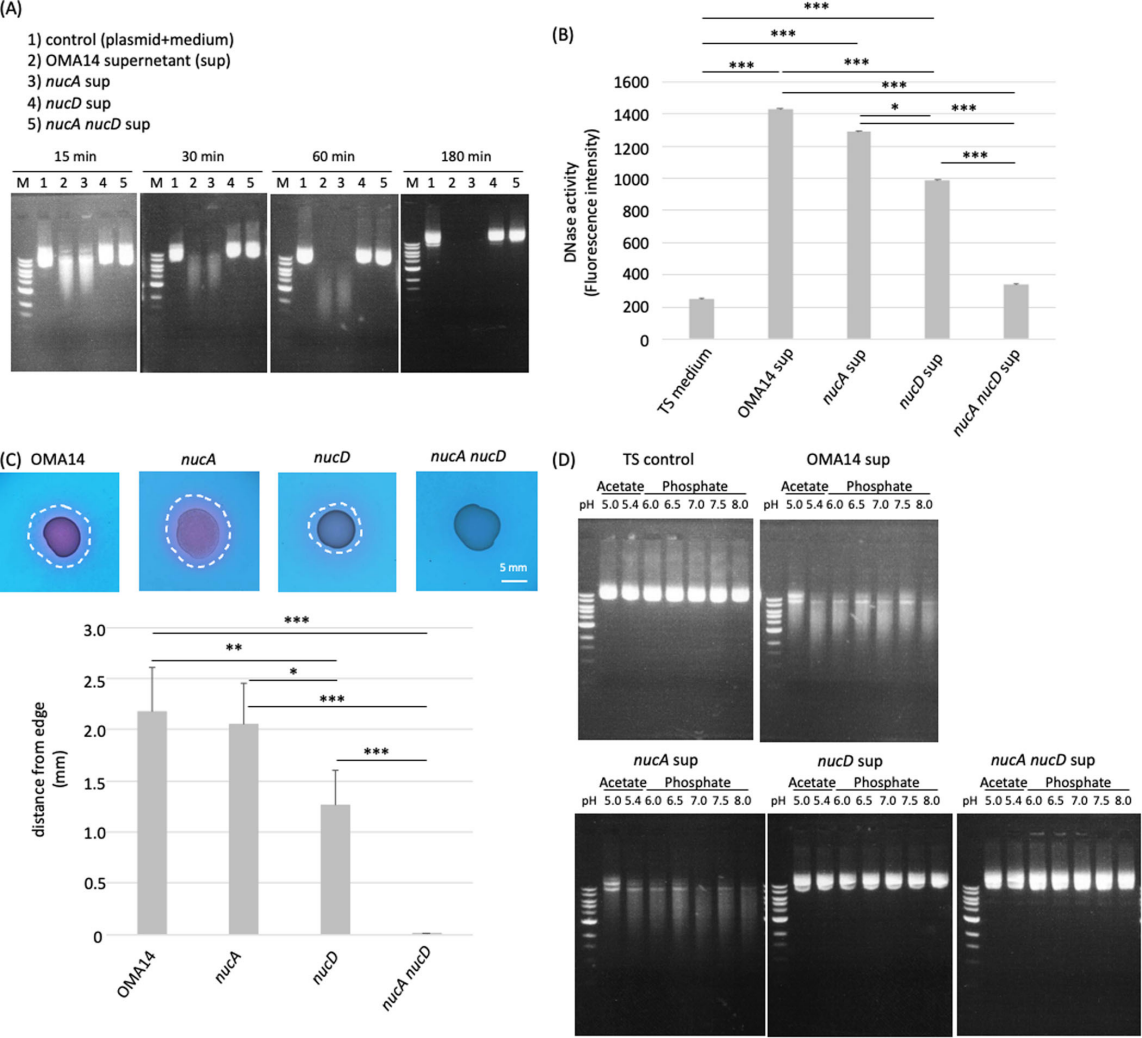
DNase activity of *P. intermedia* in vitro and in vivo. (A) DNase activity of OMA14, *nucA*, *nucD* and *nucA nucD* strains. (B) Quantitative DNase activity assay based on fluorescent intensity. Values are mean ± SD (*n* = 4). Data were analyzed using one‐way ANOVA followed by Tukey's HSD test for multiple comparisons. Asterisk marks indicate significance levels (***p < 0.001 and **p < 0.01). (C) DNase assay in vivo. Extracellular DNA degradation activity was detected on a DNA agar plate. The plates were incubated anaerobically at 37°C for 2 days. Red halo, surrounded by a dotted white circle, was observed around a colony indicated DNA degradation. Scale bar indicates 5 mm. Values are mean ± SD (*n* = 4). Data were analyzed using one‐way ANOVA followed by Tukey's HSD test for multiple comparisons. Asterisk marks indicate significance levels (***p < 0.001 and **p < 0.01). (D) *E. coli* plasmid (1 µg) was incubated for 30 min under aerobic conditions with 4 µL of TS medium or the supernants of bacterial strains (OD = 1.0) supplemented with 1 mM MgCl_2_ and 1 mM CaCl_2_ at pH 5.0–8.0.

We examined the DNase activity in the culture supernatant using *E. coli* plasmid as a substrate with an agarose gel. While the *nucD* strain did not cleave the plasmid DNA, *nucA* strain retained the activity comparable to OMA14 (Figure 2A), indicating that NucD has the major DNase activity under these conditions. When mouse genome DNA and *P. intermedia* genome DNA were also used as substrates, and identical results were obtained (data not shown). We also examined the DNase activity using Fluorescence (FL)‐based assay. The FL intensity was 10% decreased from OMA14 strain in *nucA* strain, although there was no significance in statistical analysis. In *nucD* strain, the FL intensity was 30% lower than that of OMA14 strain. However, 70% decrease in the activity was recognized in the *nucA nucD* strain, indicating the contribution of both genes in the DNase activities of the supernatant. The 30% activity remaining in the double knock out strain might be due to the contribution of other genes for DNases. Accordingly, the present gene disruption experiment indicated that major, if not all, DNase activities exerted in *P. intermedia* are encoded by these two genes.

We here examined the DNA degradation activity of *P. intermedia* using agarose DNA plate. Red zone surrounding colonies implicated the DNA degradation by extracellular secreted DNases of OMA14 (Figure 2C). *nucA* strain still exhibited an extracellular DNase activity at only 7% deceased distance to that of OMA14. In contrast, *nucD* strain showed the activity at 40% smaller area and *nucA nucD* strain did not show an extracellular DNase activity. NucD was dominant in the extracellular DNase activity under the conditions, although both of them were secreted and possessed the activity. Moreover, it should be noted that the double knockout strain scarcely showed an extracellular DNase activity.

A previous study reported that both recombinant NucA and recombinant NucD possessed pH optimum at around 5–7 (Doke et al. 2017). We examined the pH dependence of these knockout strains. The results of OMA14 and *nucA* strains supernatant showed a less efficient degradation at pH 5.0, which indicated that *nucA* strain had the broad activity at pH 5.4– 2D). The pH profile of *nucD* strain was not defined under these conditions.

Considering the results of Figure 2, we summarized the DNase activities of NucA and NucD as follows: NucA and NucD were extracellularly secreted, because they have signal sequence and their activities were detected in the culture supernatant and outside of the cell on an agar plate. Both of them possessed the DNase activity, because the most significant effect was consistently abolished in the double knockout strain in plasmid DNA assay and FL based substrate assay. The activity of NucD was predominant in an assay using plasmid and genome DNA as substrates and agar plate analysis, and fluorescent HEX‐BHQ1 substrate.

### 3.3 Biofilm Formation of *P. Intermedia* OMA14 Mutant Strains

**Figure 3:**
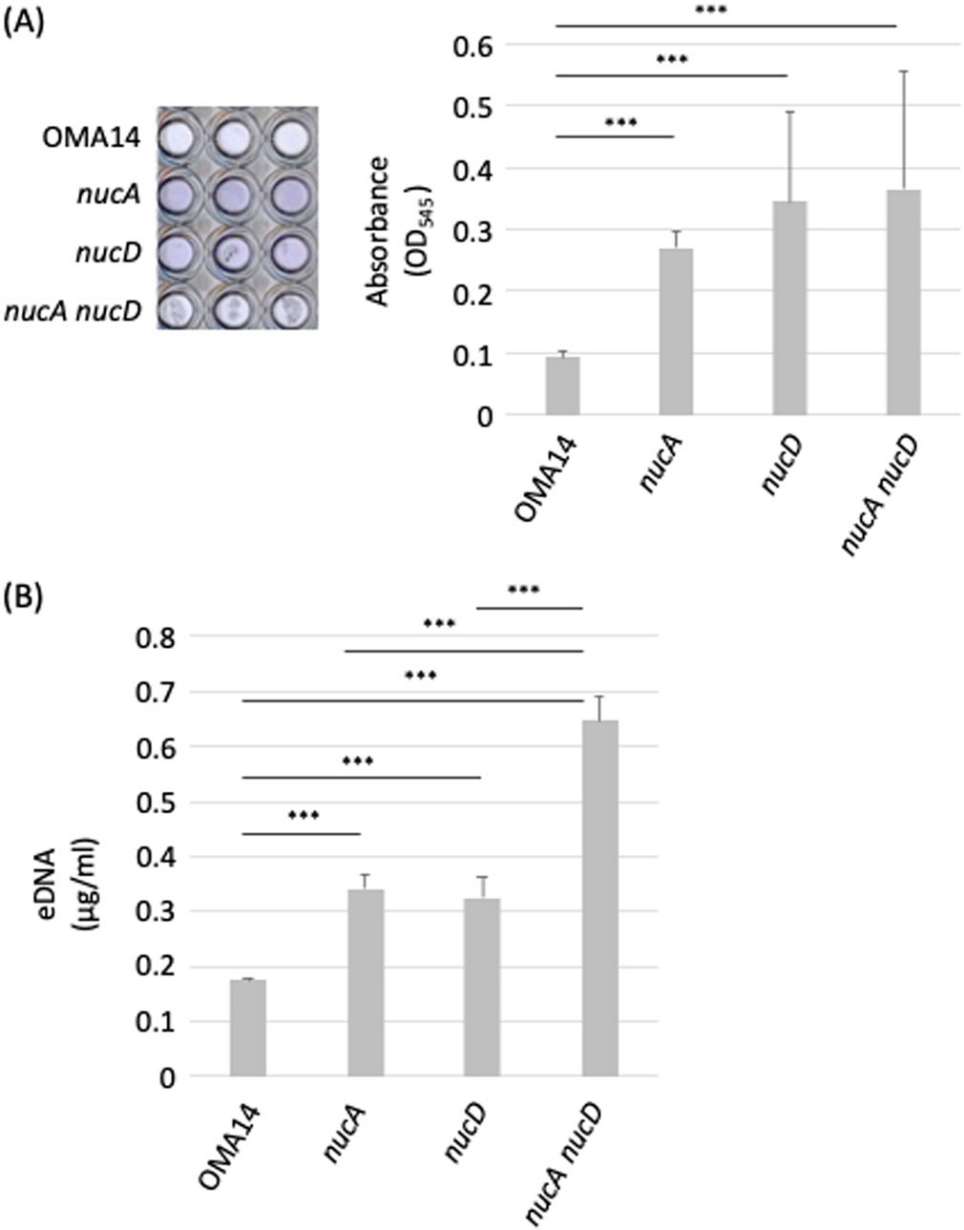
DNase is related to biofilm formation potential. (A) Biofilm formation of all *P. intermedia* strains were measured. *P. intermedia* strains were cultured in static conditions in BHI media anaerobically at 37°C for 24 h. Subsequently, quantitative measurement of biofilm was performed using the standard crystal violet‐based assay. The bar diagrams depict the Mean ± SD (*n* = 4) of the absorbance (OD_545_) values. Data were analyzed using one‐way ANOVA followed by Tukey's HSD test for multiple comparisons. Asterisks marks indicate significance levels (***p < 0.001). (B) eDNA in biofilm of all *P. intermedia* strains were measured. using PicoGreen assay. Values are mean ± SD (*n* = 3). Data were analyzed using one‐way ANOVA followed by Tukey's HSD test for multiple comparisons. Asterisk marks indicate significance levels (***p < 0.001).

Extracellular DNA (eDNA) is one of the components of biofilms. It is reported an addition of DNA‐degrading enzymes inhibited biofilm formation by *Pseudomonas aeruginosa*, indicating the possibility that eDNA contributes to biofilm formation (Whitchurch et al. 2002). Therefore, eDNA of pathogenic bacteria has also attracted attention as a target for preventing biofilm formation (Panlilio and Rice 2021). The potent DNA‐degrading activity characteristic of *P. intermedia* might decrease its own biofilm formation ability (Doke et al. 2017). Hence, we examined the possibility that the DNase activity destroies their own biofilm formation. As a result, the biofilm formation was increased in the order of *nucA*, *nucD*, and *nucA nucD* strains (Figure 3A). Directly quantify of eDNA using PicoGreen, we observed consistently that eDNA quantification revealed higher levels of eDNA in *nucA*, *nucD*, and *nucA nucD* strains than OMA14 (Figure 3B). Biofilm formation was slightly more promoted by *nucD* than *nucA* strain, while the quantity of eDNA was significantly more increased *nucA nucD* than *nucA* and *nucD* strain. The slight difference observed between the results for biofilm formation (Figure 3A) and eDNA (Figure 3B).

Taken together the results of Figures 2 and 3, *nucA* and *nucD* gene products have DNase activity, and the expression of DNase activity appeared to be varied depending on the conditions or measuring techniques.

### 3.4 Degradation of NETs

Neutrophils play an important role at the front line of immunity against infection. It has been reported that neutrophils induce a type of cell death called NETosis and release their own chromatin DNA together with neutrophil elastase (NE) and myeroperoxidase (MPO) in granules to physically capture bacteria and kill them. This mesh‐like structure is called NETs (Brinkmann et al. 2004). NETs are degraded by nucleases with releasing NETs degradation products such as cell free DNA, citH3, NE and NE‐DNA complexes. NETs remnants are eventually engulfed and degraded by phagocytes, like macrophages and dendritic cells (Brinkmann et al. 2004; Papayannopoulos 2018). It is known that NETs are induced heterogeneously, and quantitative analysis of NETs only using fluorescent imaging is difficult. We investigated the formation and degradation of NETs by biochemically quantifying the NE activity. NE is a serine protease normally expressed in neutrophil primary granules (Huang et al. 2020). NE is required for NETs formation through proteolysis of nuclear proteins leading to chromatin decondensation (Huang et al. 2020; Kolaczkowska et al. 2015; Papayannopoulos et al. 2010). On the other hand, NET degradation also releases toxic NETs degradation products such as NE and MPO that induce tissue injury and inflammation (Schauer et al. 2014).

**Figure 4:**
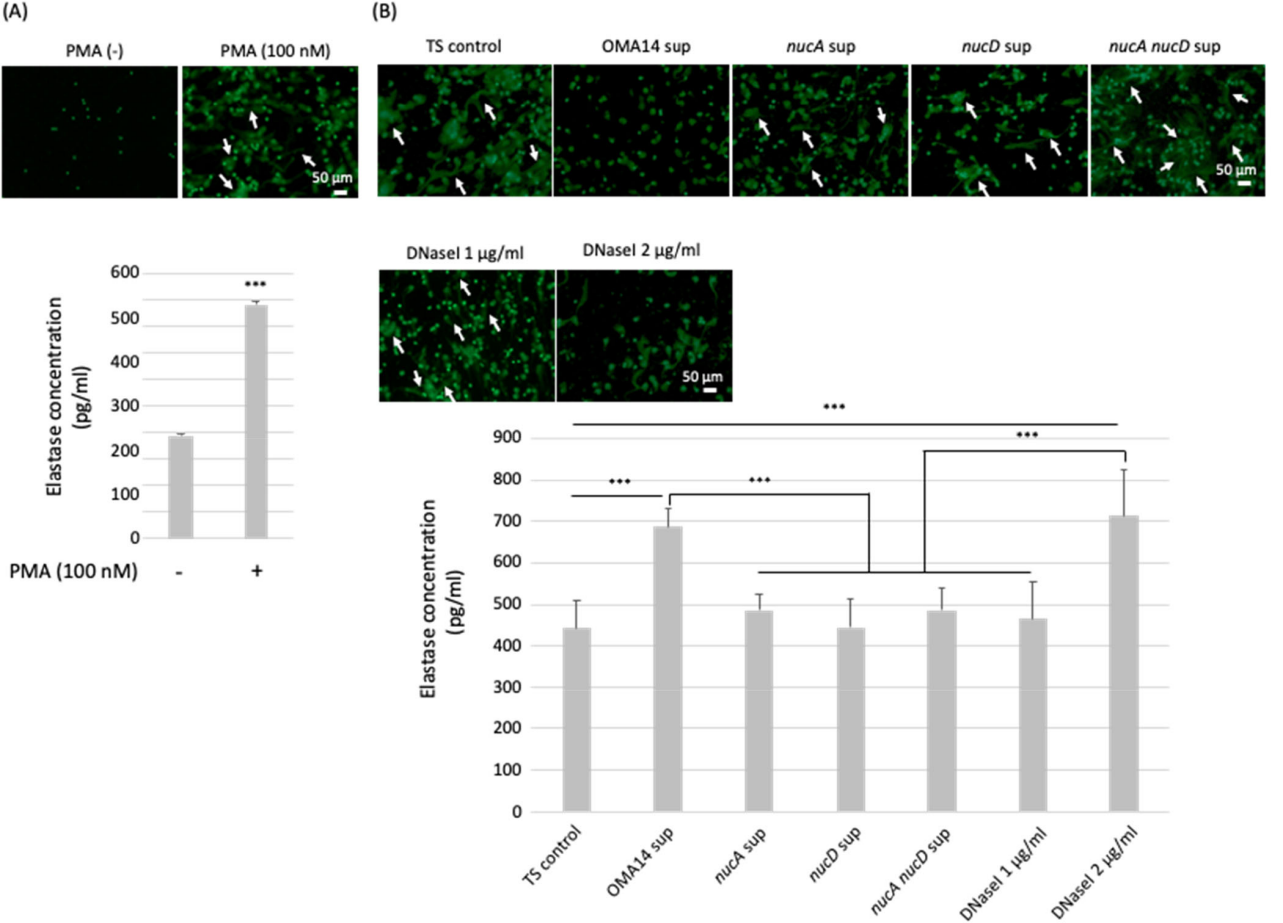
NETs degradation by *P. intermedia.* (A) The DNA in the extracellular space was stained using SYTOX green nucleic acid stain. White arrow indicates the extracellular DNA released from neutrophils. Values are mean ± SD (*n* = 4). Data were analyzed using unpaired Student's *t*‐tests. Asterisk marks indicate significance levels (***p < 0.001). (B) The DNA in the extracellular space was stained using SYTOX green nucleic acid stain. White arrow indicates the extracellular DNA released from neutrophils. Values are mean ± SD (*n* = 8). Data were analyzed using one‐way ANOVA followed by Tukey's HSD test for multiple comparisons. Asterisk marks indicate significance levels (***p < 0.001).

Comparing the PMA (‐) with PMA (100 nM) samples, PMA (100 nM) stimulation induced NETs formation (Figure 4A). NETs formation was degraded by DNase (Figure 4B: OMA14). *nucA*, *nucD* and *nucA nucD* strains decrease the ability of degrading NETs compared to OMA14. Consistent with these results, NE increased in OMA14 supernatant and decreased in *nucA*, *nucD* and *nucA nucD* strains (Figure 4B).

## 4 Discussion

*P. intermedia* is one of the main pathogenic bacteria of chronic periodontitis and exists subgingival pockets and secrets potent DNA‐degrading enzymes. However, the relationship between the DNase activity and the pathogenicity in this bacterium remains unknown. In this study, we produced gene‐disrupted strains of DNase genes of *P. intermedia* and investigated their biochemical properties, such as pH dependency, biofilm formation, and affection to NETs. *nucD* as well as OMA14 strain exhibited a broad activity at pH 5.4–8.0, which coincided with the results of recombinant forms (Doke et al. 2017), indicating that at least *nucA* and presumably *nucD* are working in the periodontal region. Socransky et al. (Socransky et al. 1998) proposed the pyramid composed of subgingival plaque based on their relationships with the severity of periodontal disease and put most pathogenic bacteria to the top of the pyramid. *P. intermedia* is located in the second step of pyramid (Orange complex) and is classified as an important bacterium in the initiation of periodontal disease. *Prevotella* spp. exists initial communities of subgingival plaque, and ensures a favorable anaerobic environment for the growth other anaerobic bacteria until the formation of biofilm (Ammann et al. 2012; Diaz et al. 2006). Biofilms are composed of various extracellular components, including eDNA, RNA, polysaccharides and proteins (Campoccia et al. 2021; Chiba et al. 2022; Kamwouo et al. 2025; Mugunthan et al. 2023). Because it has been reported that eDNA contributes to the biofilm formation, and that *P. intermedia* secrets the potent DNA degradation activity, it seems reasonable to postulate that the secreted DNase of *P. intermedia* modulates its own biofilm formation. This study revealed that both the *nucA* and *nucD* gene products appear prevent biofilm formation. NucD had higher potentials than NucA in plasmid DNA degradation (Figure 2A), FL‐based DNase quantification (Figure 2B), and agarose DNA plate experiments (Figure 2C). In comparing Figure 2A and Figure 2B, the activity of the *nucD* strain (i.e., NucA activity) is almost nonexistent in Figure 2A, whereas the activity of the *nucD* strain (i.e., NucA activity) retains 70% in Figure 2B. It has been reported that bacterial nucleases such as YhcR, NucA, and NucB are known to degrade both dsDNA and ssDNA, but their efficiency varies depending on the substrate type (Lander et al. 2024; Provvedi et al. 2001; van Sinderen et al. 1995). Therefore, the discrepancy between the plasmid degradation assay (dsDNA) (Figures 2A,C, and 3) and the fluorescence assay (ssDNA) (Figure 2B) may reflect differences in substrate sensitivity. These reasons may affect this contradiction.

Biofilms have antimicrobial resistance and immune system evasion (Lamont et al. 2018; Marsh 2005), which contribute to the chronicity and pathogenicity of periodontal disease. However if they mature too much, the environment is said to deteriorate (Prince and Jones 2023; Spormann 2008). *P. intermedia* may partially degrade biofilms with its own DNase (Figure 3A), allowing cells released from the biofilm to form new biofilms (Kaplan 2010; Tetz et al. 2009; Whitchurch et al. 2002).

NETs degrade virulence factors and kill bacteria, and DNA is a major component of NETs (Brinkmann et al. 2004). The characteristic of *Prevotella* spp., rather than their pathogenicity, might be worsening concurrent pathogen infections, as demonstrated by the mechanism of NET degradation by the bacterial nucleases (Larsen 2017). *P. intermedia* metabolites have been shown to induce severe neutrophilic inflammation in several preclinical studies (Nagaoka et al. 2014). Hence, we investigated whether the DNA degradation activity of *P. intermedia* OMA14 destroy NETs formation. NETs are degraded by endogenous DNase (Brinkmann et al. 2004), as well as nucleases produced by pathogens to evade capture by NETs (Schultz et al. 2022). OMA14 affects NETs formation. *P. intermedia* OMA14 degraded the NETs formation and DNase mutant strains retained NETs. Periodontal disease progresses through interactions with various periodontal disease‐causing bacteria present in the oral cavity. It has been suggested that *P. intermedia* degrades NETs using secreted DNases, allowing not only *P. intermedia* but also other bacteria to evade the immune system and affect the progression of periodontal disease. These findings indicate that both NucA and NucD contribute to NETs degradation in vitro, suggesting their potential involvement in evading host immune responses. However, validation in in vivo models is required to confirm their roles under physiological conditions.

This study supports findings from other periodontal pathogens. For instance, in *Porphyromonas gingivalis*, nucleases contribute to NETs degradation and immune evasion (Hajishengallis 2011; Magán‐Fernández et al. 2020; Palmer et al. 2012). Similar mechanisms have been identified in *Treponema denticola*, where extracellular nucleases disrupt host immune responses (Magán‐Fernández et al. 2020; Palmer et al. 2012). The findings in *P. intermedia* further strengthen this paradigm. It is reported that NETs are not only involved in defense against infection, but also involved in cancer, coagulation, vascular occlusion and thrombosis and formation of osteoclasts (Kong et al. 2023; Numazaki et al. 2024; Papayannopoulos 2018). Accordingly, there is a possibility that *P. intermedia* DNases is further related to such events.

There are several limitations in the present study. At present, we have not succeeded in creation of complementation strain of *nucA* and *nucD*. However, in this study, we constructed the mutant strains in a way that would minimize potential polar effects as much as possible. Because the *nucA* gene is followed immediately downstream by other genes, we constructed an insertion mutation. The native promoter region was left intact, and a termination‐less *ermF* cassette was inserted, thereby reducing the likelihood of downstream transcriptional disruption. For the *nucD* gene, the nearest downstream gene potentially subject to polar effects is located more than 500 bp away; thus, such effects are unlikely. This study exclusively utilized neutrophils isolated from 8‐week male C57BL/6 mice, which represents a key limitation. Previous reports have documented that both age and sex significantly influence NETs formation and DNase sensitivity. For example, several studies have reported that both age and sex significantly influence NETs formation and DNase sensitivity. Aging has been associated with delayed and attenuated NETs release, while sex‐based differences have been shown to modulate NETs responses (Ishikawa et al. 2024; Moreno de Lara et al. 2023). NETs were not directly detected under the in vivo model. Therefore, the findings presented here might not be generalized across sexes, age groups, or species. Future studies should include neutrophils from animals of varying ages and both sexes, as well as human‐derived neutrophils, to comprehensively assess the variability in NETs release and DNase sensitivity. The clearance of these issues should be needed to elucidate the roles of NucA and NucD in these phenomena.

In conclusion, this study revealed that the *nucA* and *nucD* gene of *P. intermedia* works as secreted DNase and that disruption mutants of DNA‐degradation enzymes modulate biofilm formation and NETs degradation. This study provides a stepping stone to clarify the effects of *P. intermedia*‐secreting DNase on *P. intermedia* itself and other periodontal pathogens. This study may be clinically relevant in the future, such as the development of new methods for preventing and treating periodontal disease.

## Author Contributions

**Fumi Seto‐Tetsuo:** conceptualization; methodology; investigation; formal analysis; supervision; project administration; resources; writing – original draft; writing – review and editing; visualization; data curation; funding acquisition; validation. **Hiroki Ashizawa:** investigation; writing – review anf editing; methodology; validation. **Yuko Sasaki:** investigation; writing – review and editing; validation. **Hideharu Yukitake:** investigation; writing – review anf editing; validation. **Mikio Shoji:** investigation; conceptualization; supervision; writing – review and editing. **Naoki Iwanaga:** writing – review and editing; supervision; validation. **Hiroshi Mukae:** writing – review and editing; supervision; validation. **Mariko Naito:** writing – review and editing; conceptualization; funding acquisition; supervision; resources; methodology; investigation; writing – original draft; project administration; validation.

## Ethics Statement

This study was approved by Nagasaki University (2109091745), and all animal experiments were performed in accordance with the guidelines of the Research Center for Biomedical Models and Animal Welfare at Nagasaki University.

## Conflicts of Interest

The authors declare no conflicts of interest.

## Declaration of Generative AI and AI‐Assisted Technologies in the Writing Process

During the preparation of this study the author used Paperpal to improve the English language and grammar of the manuscript. After using this tool, the author reviewed and edited the content as needed and takes full responsibility for the content of the publication.

## Supporting information


**Supplementary Table S1:** List of bacterial strains used in this study.
**Supplementary Table S2:** List of plasmids used in this study.
**Supplementary Table S3:** List of primers used in this study.
**Supplementary Table S4:** Statistical analysis.

## Data Availability

The data that support the findings of this study are available in Role of OxyR in Prevotella intermedia at https://www.ncbi.nlm.nih.gov/geo/query/acc.cgi?acc=GSE168003, reference number GSE168003. These data were derived from the following resources available in the public domain: ‐ NCBI GEO, https://www.ncbi.nlm.nih.gov/geo/query/acc.cgi?acc=GSE168003. The gene expression profile of the wild type was determined by transcriptome sequencing (RNA‐seq; Gene Expression Omnibus, reference number GSE168003) (Naito et al. 2021) https://www.ncbi.nlm.nih.gov/geo/query/acc.cgi?acc=GSE168003.
